# A time domain characterization of vector-valued subspace weak Gabor bi-frames

**DOI:** 10.1186/s13660-018-1733-8

**Published:** 2018-06-22

**Authors:** Jing Zhao, Yun-Zhang Li

**Affiliations:** College of Applied Sciences, Beijing University of Technology, Beijing, P.R. China

**Keywords:** 42C40, 42C15, Weak Gabor bi-frame, Vector-valued frame, Vector-valued subspace frame

## Abstract

The construction of bi-frames is a fundamental problem in frame theory. Due to their wide applications, the study of vector-valued frames and subspace frames has interested many mathematicians in recent years. In this paper, we introduce the weak Gabor bi-frame (WGBF) in vector-valued subspaces, characterize WGBFs on the time domain, and present some examples.

## Introduction

The concept of frame was introduced by Duffin and Schaeffer in [1], which dealt with nonharmonic Fourier series, and Gabor frames date back to [2]. Nowadays Gabor frames have been widely applied in signal processing [3–6]. They have been studied quite extensively on the whole space $L^{2}(\Bbb {R})$. Vector-valued frames were also called superframes, they were introduced in [7] by Han and in [8, 9] by Balan in the context of signal multiplexing, which means encoding several signals as a single one with the purpose of sharing a communication channel. In some structured cases with orthogonal windows known as superframes it is possible to find a Nyquist rate defining a phase transition between super-Riesz sequences and superframes. This was done in [10, 11] for the Gabor case, and in [12] for the wavelet case, following the constructions of vector-valued wavelet transforms [12–14]. In applications, when signals belong to a subspace of $L^{2}(\Bbb {R}, \Bbb {C}^{L})$, one would like to perform a Gabor analysis of the signals in the most efficient way, while still preserving all the features of the observed data. That is why subspace Gabor analysis has interested many mathematicians. The literature [10, 11, 15–34] has considered Gabor frames for $L^{2}(\Bbb {R}, \Bbb {C}^{L})$ and their subspaces.

We denote by $\Bbb {Z}$ the set of integers, and by $\Bbb {N}$ the set of positive integers. Let $\mathcal {H}$ be a separable Hilbert space, and $\{e_{i}\}_{i\in \mathcal {I}}$ be an at most countable sequence in $\mathcal {H}$. The sequence $\{e_{i}\}_{i\in \mathcal {I}}$ is called a *frame* (*tight frame*; *Parseval frame*) for $\mathcal{H}$ if there exist positive constants *A* and *B* such that

$$ A\Vert f \Vert ^{2}\le \sum_{i \in \mathcal {I}} \bigl\vert \langle f, e_{i} \rangle \bigr\vert ^{2}\le B \Vert f \Vert ^{2} $$

for $f\in {\mathcal{H}}$ ($A=B$; $A=B=1$); and it is called a *Bessel sequence* in $\mathcal{H}$ if the right-hand side inequality holds. Let $\{e_{i}\}_{i\in {\mathcal{I}} }$ be a frame for $\mathcal{H}$. A frame $\{\tilde{e}_{i}\}_{i\in {\mathcal{I}} }$ for $\mathcal {H}$ is called a *dual* of $\{e_{i}\}_{i\in {\mathcal{I}} }$ if

1 $$\begin{aligned} f=\sum_{i \in {\mathcal{I}}} \langle f, \tilde{e}_{i}\rangle e_{i} \quad \mbox{ for } f\in { \mathcal{H}}. \end{aligned}$$

By a simple argument, we see that (1) is equivalent to

$$\begin{aligned} f= \sum_{i \in {\mathcal{I}}} \langle f, e_{i}\rangle \tilde{e} _{i}. \end{aligned}$$

So, in this case, we also say ($\{e_{i}\}_{i\in {\mathcal{I}} }$, $\{\tilde{e}_{i}\}_{i\in {\mathcal{I}} }$) is a *pair of dual frames* (*bi-frame*) for $\mathcal{H}$. And by a standard argument, ($\{e _{i}\}_{i\in {\mathcal{I}} }$, $\{\tilde{e}_{i}\}_{i\in {\mathcal{I}} }$) is a bi-frame for $\mathcal{H}$ if and only if $\{e_{i}\}_{i\in {\mathcal{I}} }$ and $\{\tilde{e}_{i}\}_{i\in {\mathcal{I}} }$ are Bessel sequences, and

2 $$ \langle f, g\rangle = \sum_{i \in {\mathcal{I}}} \langle f, \tilde{e}_{i}\rangle \langle e_{i}, g\rangle \quad \mbox{for } f,g\in {\mathcal{H}}. $$

Given $L\in \Bbb {N}$ and *a*, $b>0$, let $L^{2}(\Bbb {R}, \Bbb {C}^{L})$ be the Hilbert space of vector-valued square integrable functions endowed with the inner product

$$\begin{aligned} \langle \mathbf{f},\tilde{\mathbf{f}}\rangle =\sum_{l=1}^{L} \int_{\mathbb{R}}f_{l}(t)\overline{{\tilde{f}}_{l}(t)} \,dt \end{aligned}$$

for $\mathbf{f}=(f_{1}, f_{2},\ldots ,f_{L}),\tilde{\mathbf{f}}=( \tilde{f}_{1}, \tilde{f}_{2},\ldots ,\tilde{f}_{L})\in L^{2}( \mathbb{R}, \mathbb{C}^{L})$. The *modulation operator* $E_{mb}$ and *translation operator* $T_{na}$ on $L^{2}(\Bbb {R}, \Bbb {C}^{L})$ with *m*, $n\in \Bbb {Z}$ are defined by

$$\begin{aligned} E_{mb}\mathbf{f}=(E_{mb}f_{1}, E_{mb}f_{2},\ldots ,E_{mb}f_{L})\quad \mbox{and}\quad T_{na}\mathbf{f}=(T_{na}f_{1}, T_{na}f_{2},\ldots ,T_{na}f_{L}) \end{aligned}$$

for $\mathbf{f}\in L^{2}(\Bbb {R}, \Bbb {C}^{L})$, respectively, where

$$\begin{aligned} E_{mb}f(\cdot )=e^{2\pi imb\cdot }f(\cdot )\quad \mbox{and}\quad T_{na}f(\cdot )=f(\cdot -na) \end{aligned}$$

for $f\in L^{2}(\Bbb {R})$. Obviously, $L^{2}(\Bbb {R}, \Bbb {C}^{L})$ is the *L*-fold direct sum of $L^{2}(\Bbb {R})$. Throughout this paper, $f_{l}$ denotes the *l*th component of $\mathbf{f}\in L^{2}(\Bbb {R}, \Bbb {C} ^{L})$, and $\mathcal{G} (\mathbf{g}, a, b)$ denotes the Gabor system:

3 $$ \mathcal{G} (\mathbf{g}, a, b)= \{ E_{mb}T_{na} \mathbf{g}: m, n\in \Bbb {Z} \} $$

for $\mathbf{g}\in L^{2}(\Bbb {R}, \Bbb {C}^{L})$. A set *S* in $\Bbb {R}$ with positive measure is said to be $a\Bbb {Z}$-*periodic* if $S+an=S$ for $n\in \Bbb {Z}$. For such *S*, we denote by $L^{2}(S, \Bbb {C}^{L})$ the closed subspace of $L^{2}(\Bbb {R}, \Bbb {C}^{L})$ of the form

$$\begin{aligned} L^{2} \bigl(S, \Bbb {C}^{L} \bigr)= \bigl\{ \mathbf{f}\in L^{2} \bigl(\Bbb {R}, \Bbb {C}^{L} \bigr): \mbox{supp}(\mathbf{f}) \subset S \bigr\} . \end{aligned}$$

For simplicity, we write $L^{2}(S)=L^{2}(S, \Bbb {C}^{1})$. As pointed out in [23], Gabor analysis on $L^{2}(S, \Bbb {C}^{L})$ with *S* being $a\Bbb {Z}$-periodic might be suitable to treat periodic and intermittent vector-valued signals.

Li and Jia in [30] introduced and characterized scalar-valued weak Gabor bi-frames (WGBFs) under the setting of subspaces of $L^{2}(\Bbb {R})$. Tian and Li in [33] characterized Gabor bi-frames on time domain in the context of vector-valued subspaces. This paper addresses vector-valued subspace weak Gabor bi-frames under the following general setup:

*General setup*:

(i) $L\in \Bbb {N}$, *a*, $b>0$;
(ii) *S* is an $a\Bbb {Z}$-periodic subset of $\Bbb {R}$.

We introduce the WGBF under the setting of vector-valued subspces, characterize WGBFs on the time domain, and also provide some examples of WGBFs. Theorem 3.1 in [30] dealt with WGBFs in scalar-valued subspaces of $L^{2}(\Bbb {R})$. Our result is a nontrivial generalization of [30], Theorem 3.1. This is because the inner product has a more complicated geometry in $L^{2}(\Bbb {R}, \Bbb {C}^{L})$ than in $L^{2}(\Bbb {R})$.

Section 2 states the main result and some related remarks. Section 3 focuses on some lemmas, which is an auxiliary one. Section 4 is devoted to proving the main result. Some examples are provided simultaneously. Finally, we give our conclusions.

## Results and discussion

### Definition 2.1

Let *L*, *a*, *b* and *S* be as in the general setup, and $\bf g$, $\mathbf{h}\in L^{2}(S, \Bbb {C}^{L})$. We say that

4 $$ \bigl(\mathcal{G}(\mathbf{g}, a, b), \mathcal{G}(\mathbf{h}, a, b) \bigr) $$

is a *weak Gabor bi-frame* (*WGBF*) for $L^{2}(S, \Bbb {C} ^{L})$ if there exists a dense subset $\mathcal{M}$ of $L^{2}(S, \mathbb{C}^{L})$ such that

5 $$\begin{aligned} \langle \mathbf{f},\tilde{\mathbf{f}}\rangle =\sum _{m,n\in \mathbb{Z}}\langle \mathbf{f}, E_{mb}T_{na} \mathbf{g}\rangle \langle E_{mb}T_{na}\mathbf{h},\tilde{ \mathbf{f}} \rangle\quad \mbox{for } \mathbf{f},\tilde{\mathbf{f}}\in \mathcal{M}, \end{aligned}$$

where the series is absolutely convergent. To be specific, in this case, we say that (4) is a *weak Gabor bi-frame* (*WGBF*) for $L^{2}(S, \Bbb {C}^{L})$ related to $\mathcal{M}$.

### Remark 2.1

Definition 2.1 reduces to [30], Definition 1.1, if $L=1$, where the authors dealt with the WGBFs for $L^{2}(S)$. In Definition 2.1, if $\mathcal{G}(\mathbf{g}, a, b)$ and $\mathcal{G}( \mathbf{h}, a, b)$ are Bessel sequences in $L^{2}(S, \Bbb {C}^{L})$, then (4) is a Gabor bi-frame (*GBF*) for $L^{2}(S, \Bbb {C} ^{L})$ by a standard argument. So WGBF generalizes GBF. In applications, sometimes we only need to consider a certain class of signals (say the signals in $\mathcal{M}$) which is dense in $L^{2}(S, \Bbb {C}^{L})$. Mathematically, it is roughly enough that (5) holds, no matter whether $\mathcal{G}(\mathbf{g}, a, b)$ and $\mathcal{G}(\mathbf{h}, a, b)$ are Bessel sequences. In this case, we can think

$$ \mathbf{f} =\sum_{m,n\in \mathbb{Z}}\langle \mathbf{f}, E_{mb}T _{na} \mathbf{g}\rangle E_{mb}T_{na}\mathbf{h} =\sum_{m,n\in \mathbb{Z}} \langle \mathbf{f}, E_{mb}T_{na} \mathbf{h} \rangle E_{mb}T_{na}\mathbf{g}\quad \mbox{for } \mathbf{f} \in \mathcal{M}. $$

### Remark 2.2

If $\mathcal {M}$ is a linear subspace of $L^{2}(S, \Bbb {C}^{L})$, Eq. (5) is equivalent to

$$ \Vert \mathbf{f} \Vert ^{2}=\sum_{m,n\in \mathbb{Z}} \langle \mathbf{f}, E_{mb}T_{na}\mathbf{g}\rangle \langle E_{mb}T_{na}\mathbf{h}, \mathbf{f}\rangle \quad \mbox{for } { \mathbf{f}}\in \mathcal {M} $$

with the right-hand side series converging absolutely by the polarization identity of inner products.

Define the set $L_{c}^{\infty }(S, \mathbb{C}^{L})$ as

$$\begin{aligned} & \bigl\{ \mathbf{f}=(f_{1}, f_{2},\ldots ,f_{L}): f_{l}\in L^{\infty }( \mathbb{R}), \\ &\quad {}{f}_{l} \mbox{ is compactly supported,} \mbox{ and } \operatorname{supp}({f}_{l}) \subset S\mbox{ for each }1\leq l\leq L \bigr\} . \end{aligned}$$

Obviously, $L_{c}^{\infty }(S, \mathbb{C}^{L})$ is a dense subspace of $L^{2}(S, \mathbb{C}^{L})$. The following theorem is our main result, it characterizes WGBFs for $L^{2}(S, \Bbb {C}^{L})$ related to $L_{c}^{\infty }(S, \Bbb {C}^{L})$ on the time domain.

### Theorem 2.1

*Let* $L\in \Bbb {N}$, *a*, $b>0$, *and* *S* *be an* $a\Bbb {Z}$-*periodic subset of* $\Bbb {R}$. *Suppose* **g**, $\mathbf{h}\in L^{2}(S, \mathbb{C} ^{L})$. *Then* $(\mathcal{G}(\mathbf{g}, a, b), \mathcal{G}( \mathbf{h}, a, b))$ *is a WGBF for* $L^{2}(S, \mathbb{C}^{L})$ *related to* $L_{c}^{\infty }(S, \mathbb{C}^{L})$ *if and only if*

6 $$\begin{aligned} \sum_{n\in \mathbb{Z}}\overline{g_{l} \biggl(t-na-\frac{k}{b} \biggr)}h_{j}(t-na)=b \delta_{k, 0}\delta_{l, j}\chi_{{S}}(t) \end{aligned}$$

*for* $k\in \Bbb {Z}$, $1\leq l, j\leq L$ *and a*.*e*. $t \in (0, a)$.

### Remark 2.3

Theorem 2.1 generalizes [30], Theorem 3.1, which dealt with the scalar case. It is a nontrivial generalization due to the complicated geometry of the inner product in $L^{2}(S, \Bbb {C}^{L})$. This can be seen from its proof in Sect. 4.

## Some lemmas

This section is devoted to some lemmas which are used in the following section. We denote by $L^{\infty }(\mathbb{R}, \mathbb{C}^{L})$ the Hilbert space

$$\begin{aligned} L^{\infty } \bigl(\mathbb{R}, \mathbb{C}^{L} \bigr)= \bigl\{ \mathbf{f}=(f_{1}, f_{2}, \ldots ,f_{L}): f_{l}\in L^{\infty }(\mathbb{R})\mbox{ for each }1 \leq l\leq L \bigr\} \end{aligned}$$

with the norm

$$\begin{aligned} \Vert \mathbf{f} \Vert _{\infty }=\max_{1\leq l\leq L}\Vert f_{l} \Vert _{\infty }. \end{aligned}$$

### Lemma 3.1

*Let* *L*, *b* *be as in the general setup*, *and* $\mathbf{g},\mathbf{h}\in L^{2}(\Bbb {R}, \Bbb {C}^{L})$. *Suppose* $\bf f$, $\tilde{\mathbf{f}} \in L^{2}(\Bbb {R}, {\Bbb {C}}^{L})$ *such that* $f_{l}$, ${\tilde{f}}_{l}$ *satisfying* $\sum_{k\in \Bbb {Z}}\vert f_{l}(\cdot -\frac{k}{b}) \vert ^{2}, \sum_{k\in \Bbb {Z}}\vert {\tilde{f}}_{l}(\cdot -\frac{k}{b}) \vert ^{2} \in L^{\infty } ( [0, \frac{1}{b}) ) $ *for each* $1\leq l \leq L$. *Then*

7 $$\begin{aligned} &\sum_{m\in \mathbb{Z}}\langle \mathbf{f}, E_{mb}\mathbf{g} \rangle \langle E_{mb}\mathbf{h},\tilde{ \mathbf{f}}\rangle \\ &\quad = \frac{1}{b}\sum_{1\leq l, j\leq L} \int_{\mathbb{R}} \overline{ \tilde{f}_{j}(t)}h_{j}(t) \sum_{k\in \mathbb{Z}}\overline{g_{l} \biggl(t- \frac{k}{b} \biggr)}f_{l} \biggl(t-\frac{k}{b} \biggr)\,dt \end{aligned}$$

*with the left*-*hand side series converging absolutely*.

### Proof

By the assumptions of $\bf f$, $\tilde{\mathbf{f}}$, we see that $\sum_{l=1}^{L}\sum_{k\in \mathbb{Z}}\vert f_{l}(\cdot -\frac{k}{b}) \vert ^{2}\leq M$, $\sum_{l=1}^{L}\sum_{k\in \mathbb{Z}}\vert {\tilde{f}}_{l}(\cdot -\frac{k}{b}) \vert ^{2} \leq M$ for some $0< M<\infty $ and each $1\leq l\leq L$. Then

$$\begin{aligned} &\sum_{l=1}^{L}\sum _{k\in \mathbb{Z}} \biggl\vert f_{l} \biggl(t- \frac{k}{b} \biggr)g_{l} \biggl(t-\frac{k}{b} \biggr) \biggr\vert \\ &\quad \leq \sum_{l=1}^{L} \biggl( \sum _{k\in \mathbb{Z}} \biggl\vert f _{l} \biggl(t- \frac{k}{b} \biggr) \biggr\vert ^{2} \biggr) ^{\frac{1}{2}} \biggl( \sum_{k\in \mathbb{Z}} \biggl\vert g_{l} \biggl(t-\frac{k}{b} \biggr) \biggr\vert ^{2} \biggr) ^{\frac{1}{2}} \\ &\quad \leq \Biggl( \sum_{l=1}^{L}\sum _{k\in \mathbb{Z}} \biggl\vert f _{l} \biggl(t- \frac{k}{b} \biggr) \biggr\vert ^{2} \Biggr) ^{\frac{1}{2}} \Biggl( \sum_{l=1}^{L}\sum _{k\in \mathbb{Z}} \biggl\vert g_{l} \biggl(t- \frac{k}{b} \biggr) \biggr\vert ^{2} \Biggr) ^{\frac{1}{2}} \\ &\quad \leq \sqrt{M} \Biggl( \sum_{l=1}^{L} \sum_{k\in \mathbb{Z}} \biggl\vert g_{l} \biggl(t- \frac{k}{b} \biggr) \biggr\vert ^{2} \Biggr) ^{\frac{1}{2}}. \end{aligned}$$

And thus

8 $$\begin{aligned} & \int_{0}^{\frac{1}{b}} \Biggl( \sum _{l=1}^{L}\sum_{k\in \mathbb{Z}} \biggl\vert f_{l} \biggl(t-\frac{k}{b} \biggr)g_{l} \biggl(t- \frac{k}{b} \biggr) \biggr\vert \Biggr) ^{2}\,dt \\ &\quad \leq M \int_{0}^{\frac{1}{b}}\sum_{l=1}^{L} \sum_{k\in \mathbb{Z}} \biggl\vert g_{l} \biggl(t- \frac{k}{b} \biggr) \biggr\vert ^{2}\,dt \\ &\quad \leq M\Vert \mathbf{g} \Vert ^{2} \\ &\quad < \infty . \end{aligned}$$

Similarly,

9 $$\begin{aligned} \int_{0}^{\frac{1}{b}} \Biggl( \sum _{l=1}^{L}\sum_{k\in \mathbb{Z}} \biggl\vert {\tilde{f}}_{l} \biggl(t-\frac{k}{b} \biggr)h_{l} \biggl(t- \frac{k}{b} \biggr) \biggr\vert \Biggr) ^{2}\,dt \leq M \Vert \mathbf{h} \Vert ^{2}< \infty . \end{aligned}$$

By a simple calculation, we have

$$\begin{aligned} &\langle {\mathbf{f}}, {E}_{mb}{\mathbf{g}}\rangle = \int_{0}^{ \frac{1}{b}} \Biggl( \sum _{l=1}^{L}\sum_{k\in \mathbb{Z}}f _{l} \biggl(t-\frac{k}{b} \biggr)\overline{g_{l} \biggl(t-\frac{k}{b} \biggr)} \Biggr) e^{-2\pi imbt}\,dt, \\ &\langle {{E}_{mb}{\mathbf{h}}, \tilde{\mathbf{f}}}\rangle = \int_{0} ^{\frac{1}{b}} \Biggl( \sum _{l=1}^{L}\sum_{k\in \mathbb{Z}} \overline{ \tilde{f}_{l} \biggl(t-\frac{k}{b} \biggr)}h_{l} \biggl(t-\frac{k}{b} \biggr) \Biggr) e^{2\pi imbt}\,dt, \end{aligned}$$

for $m\in \Bbb {Z}$. Again using (8) and (9), we see that the series $\sum_{m\in \Bbb {Z}}\langle {\mathbf{f}}, {E}_{mb}{\mathbf{g}}\rangle \langle {{E}_{mb}{\mathbf{h}}, \tilde{\mathbf{f}}} \rangle $ converges absolutely, and thus

10 $$\begin{aligned} &\sum_{m\in \mathbb{Z}}\langle {\mathbf{f}}, {E}_{mb}{\mathbf{g}} \rangle \langle {{E}_{mb}{\mathbf{h}}, \tilde{\mathbf{f}}}\rangle \\ &\quad =\frac{1}{b} \int_{0}^{\frac{1}{b}} \Biggl( \sum _{l=1}^{L}\sum_{k\in \mathbb{Z}}f_{l} \biggl(t-\frac{k}{b} \biggr)\overline{g_{l} \biggl(t- \frac{k}{b} \biggr)} \Biggr) \Biggl( \sum_{j=1}^{L} \sum_{k\in \mathbb{Z}}\overline{\tilde{f}_{j} \biggl(t- \frac{k}{b} \biggr)}h_{j} \biggl(t- \frac{k}{b} \biggr) \Biggr)\,dt, \end{aligned}$$

because of $\{ \sqrt{b}e^{2\pi imbt}:m\in \Bbb {Z} \} $ being an orthonormal basis for $L^{2} ( [0, \frac{1}{b}) ) $. Write

$$\begin{aligned} J(t)=\sum_{l=1}^{L}\sum _{k\in \Bbb {Z}}f_{l} \biggl(t-\frac{k}{b} \biggr) \overline{g _{l} \biggl(t-\frac{k}{b} \biggr)}. \end{aligned}$$

Then (10) is equivalent to

$$\begin{aligned} \sum_{m\in \mathbb{Z}}\langle {\mathbf{f}}, {E}_{mb}{ \mathbf{g}} \rangle \langle {{E}_{mb}{\mathbf{h}}, \tilde{\mathbf{f}}} \rangle = \frac{1}{b} \int_{0}^{\frac{1}{b}}\sum_{j=1}^{L} \sum_{k\in \mathbb{Z}}\overline{\tilde{f}_{j} \biggl(t- \frac{k}{b} \biggr)}h_{j} \biggl(t- \frac{k}{b} \biggr)J(t)\,dt. \end{aligned}$$

Observing that

$$\begin{aligned} &\sum_{j=1}^{L}\sum _{k\in \mathbb{Z}} \biggl\vert \overline{ \tilde{f}_{j} \biggl(t-\frac{k}{b} \biggr)}h_{j} \biggl(t-\frac{k}{b} \biggr)J(t) \biggr\vert \\ &\quad \leq \Biggl( \sum_{j=1}^{L}\sum _{k\in \mathbb{Z}} \biggl\vert \tilde{f} _{j} \biggl(t- \frac{k}{b} \biggr)h_{j} \biggl(t-\frac{k}{b} \biggr) \biggr\vert \Biggr) \Biggl( \sum_{l=1}^{L} \sum_{k\in \mathbb{Z}} \biggl\vert f_{l} \biggl(t- \frac{k}{b} \biggr)g_{l} \biggl(t-\frac{k}{b} \biggr)\biggr\vert \Biggr) \in L^{1} \biggl(\biggl[0, \frac{1}{b} \biggr)\biggr) \end{aligned}$$

by (8) and (9), we have

$$\begin{aligned} &\sum_{m\in \mathbb{Z}}\langle {\mathbf{f}}, {E}_{mb}{ \mathbf{g}} \rangle \langle {{E}_{mb}{\mathbf{h}}, \tilde{\mathbf{f}}} \rangle \\ &\quad =\frac{1}{b}\sum_{j=1}^{L}\sum _{k\in \mathbb{Z}} \int _{0}^{\frac{1}{b}}\overline{\tilde{f}_{j} \biggl(t-\frac{k}{b} \biggr)}h_{j} \biggl(t- \frac{k}{b} \biggr)J(t)\,dt \\ &\quad =\frac{1}{b}\sum_{j=1}^{L} \int_{\mathbb{R}}\overline{ \tilde{f}_{j}(t)}h_{j}(t)J(t) \,dt \end{aligned}$$

by the Lebesgue dominated convergence theorem. It follows that

$$\begin{aligned} &\sum_{m\in \mathbb{Z}}\langle {\mathbf{f}}, {E}_{mb}{ \mathbf{g}} \rangle \langle {{E}_{mb}{\mathbf{h}}, \tilde{\mathbf{f}}} \rangle \\ &\quad = \frac{1}{b}\sum_{1\leq l, j\leq L} \int_{\mathbb{R}}\overline{ \tilde{f}_{j}(t)}h_{j}(t) \sum_{k\in \mathbb{Z}}\overline{g _{l} \biggl(t- \frac{k}{b} \biggr)}f_{l} \biggl(t-\frac{k}{b} \biggr)\,dt. \end{aligned}$$

 □

### Lemma 3.2

*Let* *L*, *a* *and* *b* *be as in the general setup*, *and* $\mathbf{g}, \mathbf{h} \in L^{2}(\Bbb {R}, \Bbb {C}^{L})$. *Then*, *for* $\mathbf{f}, \tilde{\mathbf{f}}\in L_{c}^{\infty }(\mathbb{R}, \mathbb{C}^{L})$, *we have*

11 $$\begin{aligned} &\sum_{m,n\in \mathbb{Z}}\langle \mathbf{f}, E_{mb}T_{na} \mathbf{g}\rangle \langle E_{mb}T_{na} \mathbf{h},\tilde{\mathbf{f}} \rangle \\ &\quad =\frac{1}{b}\sum_{1\leq l, j\leq L} \int_{\mathbb{R}}\sum_{k\in \mathbb{Z}} \biggl( \sum _{n\in \mathbb{Z}}\overline{g _{l} \biggl(t-na- \frac{k}{b} \biggr)}h_{j}(t-na) \biggr) f_{l} \biggl(t- \frac{k}{b} \biggr)\overline{ {\tilde{f}}_{j}(t)}\,dt \end{aligned}$$

*with the left*-*hand side series converging absolutely*.

### Proof

Fix $\mathbf{f},\tilde{\mathbf{f}}\in L_{c}^{\infty }(\mathbb{R}, \mathbb{C}^{L})$. It is easy to check $\sum_{l=1}^{L}\sum_{k\in \mathbb{Z}}\vert f_{l}(\cdot -\frac{k}{b}) \vert ^{2}$, $\sum_{l=1}^{L}\sum_{k\in \mathbb{Z}}\vert \tilde{f}_{l}(\cdot -\frac{k}{b}) \vert ^{2}\in L^{\infty }([0, \frac{1}{b}))$. For an arbitrary $n\in \mathbb{Z}$, replace **g** and **h** in Lemma 3.1 by $T_{na}\mathbf{g}$ and $T_{na}\mathbf{h}$, respectively. Then

12 $$\begin{aligned} &\sum_{m\in \mathbb{Z}}\langle \mathbf{f}, E_{mb}T_{na} \mathbf{g}\rangle \langle E_{mb}T_{na} \mathbf{h},\tilde{\mathbf{f}} \rangle \\ &\quad =\frac{1}{b}\sum_{1\leq l, j\leq L} \int_{\mathbb{R}}\sum_{k\in \mathbb{Z}} \overline{g_{l} \biggl(t-na-\frac{k}{b} \biggr)}h_{j}(t-na) f_{l} \biggl(t-\frac{k}{b} \biggr)\overline{{ \tilde{f}}_{j}(t)}\,dt. \end{aligned}$$

Noting that $\mathcal{G}(\mathbf{f}, a, b)$ is a Bessel sequence by [24], Proposition 6.2.2, and

$$\begin{aligned} &\bigl\vert \langle \mathbf{f}, E_{mb}T_{na}\mathbf{g} \rangle \bigr\vert = \bigl\vert \langle E_{-mb}T_{-na} \mathbf{f},\mathbf{g}\rangle \bigr\vert , \\ & \bigl\vert \langle \tilde{ \mathbf{f}},E_{mb}T_{na}\mathbf{h}\rangle \bigr\vert = \bigl\vert \langle E_{-mb}T_{-na}\tilde{\mathbf{f}},\mathbf{h} \rangle \bigr\vert , \end{aligned}$$

we have $\{\langle \mathbf{f}, E_{mb}T_{na}\mathbf{g}\rangle \}_{m,n \in \mathbb{Z}}, \{\langle \tilde{\mathbf{f}}, E_{mb}T_{na} \mathbf{h}\rangle \}_{m,n\in \mathbb{Z}}\in l^{2}(\mathbb{Z}^{2})$, and thus the left-hand side series of Eq. (11) is absolutely convergent. Hence,

13 $$\begin{aligned} &\sum_{m,n\in \mathbb{Z}}\langle \mathbf{f}, E_{mb}T_{na} \mathbf{g}\rangle \langle E_{mb}T_{na} \mathbf{h},\tilde{\mathbf{f}} \rangle \\ &\quad =\sum_{n\in \mathbb{Z}}\sum_{m\in \mathbb{Z}} \langle \mathbf{f}, E_{mb}T_{na}\mathbf{g}\rangle \langle E_{mb}T_{na} \mathbf{h},\tilde{\mathbf{f}}\rangle \\ &\quad =\frac{1}{b}\sum_{1\leq l, j\leq L}\sum _{n\in \mathbb{Z}} \int_{\mathbb{R}}\sum_{k\in \mathbb{Z}} \overline{g _{l} \biggl(t-na-\frac{k}{b} \biggr)}h_{j}(t-na) f_{l} \biggl(t-\frac{k}{b} \biggr)\overline{ { \tilde{f}}_{j}(t)}\,dt \end{aligned}$$

by (12). Assume that $\operatorname{supp}(f_{l}),\operatorname{supp}({\tilde{f}}_{l})\subset [-Na, Na]$ for some $N\in \Bbb {N}$ and each $1\leq l\leq L$. Then there exists $P\in \mathbb{N}$ such that $f_{l}(t-\frac{k}{b}) \overline{{\tilde{f}}_{j}(t)}=0$ for $\vert k \vert \geq P$ and $1\leq l, j \leq L$. And thus

$$\begin{aligned} & \int_{\mathbb{R}}\sum_{n\in \mathbb{Z}}\sum _{k\in \mathbb{Z}} \biggl\vert g_{l} \biggl(t-na- \frac{k}{b} \biggr)h_{j}(t-na) f _{l} \biggl(t- \frac{k}{b} \biggr){\tilde{f}}_{j}(t) \biggr\vert \,dt \\ &\quad = \int_{-Na}^{Na}\sum_{n\in \mathbb{Z}} \sum_{\vert k \vert \leq P} \biggl\vert g_{l} \biggl(t-na- \frac{k}{b} \biggr)h_{j}(t-na) f_{l} \biggl(t- \frac{k}{b} \biggr){\tilde{f}} _{j}(t) \biggr\vert \,dt \\ &\quad \leq \Vert \mathbf{f} \Vert _{\infty }\Vert \tilde{\mathbf{f}} \Vert _{\infty } \sum_{\vert k \vert \leq P}\sum _{n\in \mathbb{Z}} \int_{-Na}^{Na} \biggl\vert g_{l} \biggl(t-na-\frac{k}{b} \biggr)h_{j}(t-na) \biggr\vert \,dt \\ &\quad =\Vert \mathbf{f} \Vert _{\infty }\Vert \tilde{\mathbf{f}} \Vert _{\infty }\sum_{\vert k \vert \leq P}\sum _{n\in \mathbb{Z}}\sum_{j=-N} ^{N-1} \int_{0}^{a} \biggl\vert g_{l} \biggl(t-(n-j)a-\frac{k}{b} \biggr)h_{j} \bigl(t-(n-j)a \bigr) \biggr\vert \,dt \\ &\quad =\Vert \mathbf{f} \Vert _{\infty }\Vert \tilde{\mathbf{f}} \Vert _{\infty }\sum_{\vert k \vert \leq P}2N\sum _{n\in \mathbb{Z}} \int_{0}^{a} \biggl\vert g _{l} \biggl(t-na-\frac{k}{b} \biggr)h_{j}(t-na) \biggr\vert \,dt \\ &\quad =2N\Vert \mathbf{f} \Vert _{\infty }\Vert \tilde{\mathbf{f}} \Vert _{\infty }\sum_{\vert k \vert \leq P} \int_{\mathbb{R}} \biggl\vert g_{l} \biggl(t- \frac{k}{b} \biggr)h_{j}(t) \biggr\vert \,dt \\ &\quad < \infty \end{aligned}$$

for $1\leq l, j\leq L$. Again using (13), we obtain

$$\begin{aligned} &\sum_{m,n\in \mathbb{Z}}\langle \mathbf{f}, E_{mb}T_{na} \mathbf{g}\rangle \langle E_{mb}T_{na}\mathbf{h},\tilde{ \mathbf{f}} \rangle \\ &\quad =\frac{1}{b}\sum_{1\leq l, j\leq L} \int_{\mathbb{R}}\sum_{k\in \mathbb{Z}} \biggl( \sum _{n\in \mathbb{Z}}\overline{g _{l} \biggl(t-na- \frac{k}{b} \biggr)}h_{j}(t-na) \biggr) f_{l} \biggl(t- \frac{k}{b} \biggr)\overline{ {\tilde{f}}_{j}(t)}\,dt. \end{aligned}$$

 □

## Proof of Theorem 2.1 and some examples

**Proof of Theorem ****2.1****.** Observe that $L_{c}^{\infty }(S, \mathbb{C}^{L})$ is a linear dense subspace of $L^{2}(S, \mathbb{C}^{L})$. It follows that (4) being a WGBF for $L^{2}(S, \mathbb{C}^{L})$ related to $L_{c}^{\infty }(S, \mathbb{C}^{L})$ is equivalent to

14 $$\begin{aligned} &\langle \mathbf{f}, \tilde{\mathbf{f}}\rangle =\frac{1}{b}\sum_{1\leq l, j\leq L} \int_{\mathbb{R}}\sum_{k\in \mathbb{Z}} \biggl( \sum _{n\in \mathbb{Z}}\overline{g _{l} \biggl(t-na- \frac{k}{b} \biggr)}h_{j}(t-na) \biggr) f_{l} \biggl(t- \frac{k}{b} \biggr)\overline{ {\tilde{f}}_{j}(t)}\,dt \end{aligned}$$

for $\mathbf{f},\tilde{\mathbf{f}}\in L_{c}^{\infty }(S, \mathbb{C}^{L})$ by Definition 2.1 and Lemma 3.2.

The proof of sufficiency is obvious. Next we will prove the necessity. Since

$$\begin{aligned} & \int_{0}^{a}\sum_{n\in \mathbb{Z}} \biggl\vert \overline{g_{l} \biggl(t-na-\frac{k}{b} \biggr)}h_{j}(t-na) \biggr\vert \,dt \\ &\quad \leq \int_{0}^{a} \biggl( \sum _{n\in \mathbb{Z}} \biggl\vert g_{l} \biggl(t-na- \frac{k}{b} \biggr) \biggr\vert ^{2} \biggr) ^{\frac{1}{2}} \biggl( \sum_{n\in \mathbb{Z}} \bigl\vert h_{j}(t-na) \bigr\vert ^{2} \biggr) ^{\frac{1}{2}}\,dt \\ &\quad \leq \biggl( \int_{0}^{a}\sum_{n\in \mathbb{Z}} \biggl\vert g_{l} \biggl(t-na-\frac{k}{b} \biggr) \biggr\vert ^{2}\,dt \biggr) ^{\frac{1}{2}} \biggl( \int_{0}^{a}\sum_{n\in \mathbb{Z}} \bigl\vert h_{j}(t-na) \bigr\vert ^{2}\,dt \biggr) ^{\frac{1}{2}} \\ &\quad =\Vert g_{l} \Vert _{L^{2}(\mathbb{R})}\Vert h_{j} \Vert _{L^{2}(\mathbb{R})}< \infty , \end{aligned}$$

almost all points in $(0, a)$ are Lebesgue points of

$$\begin{aligned} \sum_{n\in \mathbb{Z}}\overline{g_{l} \biggl(t-na- \frac{k}{b} \biggr)}h_{j}(t-na)\quad \mbox{for } 1\leq l, j\leq L, k \in \mathbb{Z}. \end{aligned}$$

Fix $1\leq l_{0}, j_{0} \leq L, k_{0}\in \mathbb{Z}$ and such a Lebesgue point $t_{0}\in (0, a)$. Take $0<\epsilon <\frac{1}{2b}$ such that $B_{\epsilon }=(t_{0}-\epsilon , t_{0}+\epsilon )\subset (0, a)$, and define $\bf f$, $\tilde{\mathbf{f}}$ in (14) by

$$\begin{aligned}& \mathbf{f}=(0,\ldots ,0, f_{l_{0}}, 0,\ldots ,0),\qquad f_{l_{0}}\biggl(t-\frac{k_{0}}{b} \biggr) =\frac{\chi_{{B_{\epsilon }}}(t)\chi_{{S}}(t)}{\sqrt{2 \epsilon }}, \\& \tilde{\mathbf{f}}=(0,\ldots ,0, \tilde{f}_{j_{0}}, 0,\ldots ,0),\qquad \tilde{f}_{j_{0}}(t) =\frac{\chi_{{B_{\epsilon }}}(t)\chi_{{S}}(t)}{\sqrt{2 \epsilon }}. \end{aligned}$$

Then

15 $$\begin{aligned} & \int_{\mathbb{R}}\frac{\chi_{{B_{\epsilon }}}(t)\chi_{{S}}(t)}{\sqrt{2 \epsilon }} \frac{\chi_{{B_{\epsilon }}}(t+\frac{k_{0}}{b})\chi_{{S}}(t+\frac{k_{0}}{b})}{\sqrt{2\epsilon }}\,dt \\ &\quad =\frac{1}{b} \int_{\mathbb{R}}\sum_{n\in \mathbb{Z}} \overline{g_{l _{0}} \biggl(t-na-\frac{k_{0}}{b} \biggr)}h_{l_{0}}(t-na) \frac{\chi_{{B_{\epsilon }}}(t)\chi_{{S}}(t)}{{2\epsilon }}\,dt \end{aligned}$$

when $l_{0}=j_{0}$, and

16 $$\begin{aligned} 0 =\frac{1}{b} \int_{\mathbb{R}}\sum_{n\in \mathbb{Z}} \overline{g_{l _{0}} \biggl(t-na-\frac{k_{0}}{b} \biggr)}h_{j_{0}}(t-na) \frac{\chi_{{B_{\epsilon }}}(t)\chi_{{S}}(t)}{{2\epsilon }}\,dt \end{aligned}$$

when $l_{0}\neq j_{0}$. From (15), we have

$$ \frac{1}{2\epsilon } \int_{B_{\epsilon }}\delta_{{k_{0}}, 0}\chi_{{S}}(t)\,dt = \frac{1}{2b\epsilon } \int_{B_{\epsilon }} \sum_{n\in \mathbb{Z}} \overline{g_{l_{0}} \biggl(t-na-\frac{k_{0}}{b} \biggr)}h_{l _{0}}(t-na) \chi_{{S}}(t)\,dt. $$

This leads to

17 $$\begin{aligned} \sum_{n\in \mathbb{Z}}\overline{g_{l_{0}} \biggl(t_{0}-na-\frac{k_{0}}{b} \biggr)}h _{l_{0}}(t_{0}-na) \chi_{{S}}(t_{0})=b\delta_{k_{0}, 0}\chi_{{S}}(t _{0}) \end{aligned}$$

by letting $\epsilon \rightarrow 0$. Because of $h_{l_{0}}\in L^{2}(S)$, (17) is equivalent to

18 $$\begin{aligned} \sum_{n\in \mathbb{Z}}\overline{g_{l_{0}} \biggl(t_{0}-na-\frac{k_{0}}{b} \biggr)}h _{l_{0}}(t_{0}-na)=b \delta_{k_{0}, 0}\chi_{{S}}(t_{0}). \end{aligned}$$

From (16), we have

$$\begin{aligned} 0 =\frac{1}{2b\epsilon } \int_{B_{\epsilon }}\sum_{n\in \mathbb{Z}}\overline{g _{l_{0}} \biggl(t-na-\frac{k_{0}}{b} \biggr)}h_{j_{0}}(t-na) \chi_{{S}}(t)\,dt. \end{aligned}$$

This leads to

19 $$\begin{aligned} \sum_{n\in \mathbb{Z}}\overline{g_{l_{0}} \biggl(t_{0}-na-\frac{k_{0}}{b} \biggr)}h _{j_{0}}(t_{0}-na) \chi_{{S}}(t_{0})=0 \end{aligned}$$

by letting $\epsilon \rightarrow 0$. Because of $h_{j_{0}}\in L^{2}(S)$, (19) is equivalent to

20 $$\begin{aligned} \sum_{n\in \mathbb{Z}}\overline{g_{l_{0}} \biggl(t_{0}-na-\frac{k_{0}}{b} \biggr)}h _{j_{0}}(t_{0}-na)=0. \end{aligned}$$

Combining (18) with (20), we have

$$\begin{aligned} \sum_{n\in \mathbb{Z}}\overline{g_{l_{0}} \biggl(t_{0}-na-\frac{k_{0}}{b} \biggr)}h_{j_{0}}(t_{0}-na)=b \delta_{k_{0}, 0}\delta_{l_{0}, j_{0}}\chi_{{S}}(t_{0}). \end{aligned}$$

Therefore, (6) holds by the arbitrariness of $j_{0}$, $l _{0}$, $k_{0}$ and $t_{0}$.

### Example 4.1

Let *L*, *a*, *b* and *S* be as in the general setup, and $ab\leq \frac{1}{2}$. Suppose **g**, $\mathbf{h}\in L^{2}(S, \mathbb{C}^{L})$, satisfy $\mathrm{{supp}}(\mathbf{g}), \mathrm{{supp}}( \mathbf{h})\subset (-a, a)$ and

21 $$\begin{aligned} \overline{g_{l}(t)}h_{j}(t)+ \overline{g_{l}(t-a)}h_{j}(t-a) =b \delta_{l, j} \chi_{{S}}(t) \end{aligned}$$

for $1\leq l, j\leq L$ and a.e. $t\in (0, a)$. Then (4) is a WGBF for $L^{2}(S, \mathbb{C}^{L})$ related to $L_{c}^{\infty }(S, \mathbb{C}^{L})$. In particular, if $\mathbf{g}, \mathbf{h}\in L ^{\infty }(\mathbb{R}, \mathbb{C}^{L})$ in addition, (4) is a GBF for $L^{2}(S, \mathbb{C}^{L})$.

### Proof

By a standard argument, we have

22 $$\begin{aligned} \sum_{n\in \mathbb{Z}}\overline{g_{l} \biggl(t-na-\frac{k}{b} \biggr)}h_{j}(t-na) = \overline{g_{l} \biggl(t-a-\frac{k}{b} \biggr)}h_{j}(t-a)+ \overline{g_{l} \biggl(t- \frac{k}{b} \biggr)}h_{j}(t) \end{aligned}$$

for $k\in \mathbb{Z}$, $1\leq l, j\leq L$ and a.e. $t\in (0, a)$. Since $ab\leq \frac{1}{2}$, we have $(-a, a)\cap ((-a, a)+ \frac{k}{b})=\emptyset $ for $0\neq k\in \mathbb{Z}$. And because of $\mbox{supp}(\mathbf{g})\subset (-a, a)$, we have

$$\begin{aligned} g_{l} \biggl(t-a-\frac{k}{b} \biggr)=g_{l} \biggl(t- \frac{k}{b} \biggr)=0 \end{aligned}$$

for $0\neq k\in \mathbb{Z}$, $1\leq l\leq L$ and a.e. $t\in (0, a)$. Using (22), we obtain

$$\begin{aligned} \sum_{n\in \mathbb{Z}}\overline{g_{l} \biggl(t-na- \frac{k}{b} \biggr)}h_{j}(t-na)=0 \end{aligned}$$

for $0\neq k\in \mathbb{Z}$, $1\leq l, j\leq L$ and a.e. $t\in (0, a)$. It follows that

$$\begin{aligned} &\sum_{n\in \mathbb{Z}}\overline{g_{l} \biggl(t-na- \frac{k}{b} \biggr)}h_{j}(t-na)=b \delta_{k, 0} \delta_{l, j}\chi_{{S}}(t) \end{aligned}$$

for $k\in \mathbb{Z}$, $1\leq l, j\leq L$ and a.e. $t\in (0, a)$ by (21). Therefore (4) is a WGBF for $L^{2}(S, \mathbb{C}^{L})$ related to $L_{c}^{\infty }(S, \mathbb{C}^{L})$ by Theorem 2.1. Furthermore, suppose $\mathbf{g}, \mathbf{h} \in L^{\infty }(\mathbb{R}, \mathbb{C}^{L})$ in addition, then $\mathbf{g}, \mathbf{h}\in L_{c}^{\infty }(\mathbb{R}, \mathbb{C} ^{L})$. Therefore, $\mathcal{G}(\mathbf{g}, a, b)$ and $\mathcal{G}( \mathbf{h}, a, b)$ are Bessel sequences by [24], Proposition 6.2.2. Then, using $\mathbf{g}, \mathbf{h}\in L^{2}(S, \mathbb{C} ^{L})$, we see that they are Bessel sequences in $L^{2}(S, \mathbb{C}^{L})$. So (4) is a GBF for $L^{2}(S, \mathbb{C} ^{L})$ by Remark 2.1. □

### Example 4.2

Let $L=2$, *a*, $b>0$ satisfying $ab\leq \frac{1}{2}$, $0<\alpha , \beta <\frac{1}{2}$, $0< c\leq a$ and $S=\bigcup_{n\in \mathbb{Z}}[(0, c)+na]$. Define $\mathbf{g}(t)=(g_{1}(t), g_{2}(t)), \mathbf{h}(t)=(h_{1}(t), h_{2}(t))$ as follows:

$$\begin{aligned} &g_{1}(t)=\chi_{(0, c)}(t)t^{\alpha }+( \overline{c_{1}} +\overline{c _{2}})\chi_{(-a, c-a)}(t) (t+a)^{-\beta }, \\ &g_{2}(t)=(\overline{c_{1}}-\overline{c_{2}}) \chi_{(0, c)}(t)t^{ \alpha } + \bigl(1+\overline{c_{1}^{2}}- \overline{c_{2}^{2}} \bigr)\chi_{(-a, c-a)}(t) (t+a)^{- \beta }, \\ &h_{1}(t)=b \bigl(1+{c_{1}^{2}}-{c_{2}^{2}} \bigr)\chi_{(0, c)}(t)t^{-\alpha } -b(c _{1}-c_{2}) \chi_{(-a, c-a)}(t) (t+a)^{\beta } , \\ &h_{2}(t)=-b(c_{1}+c_{2})\chi_{(0, c)}(t)t^{-\alpha }+b \chi_{(-a, c-a)}(t) (t+a)^{ \beta }, \end{aligned}$$

where $c_{1}$ and $c_{2}$ are two complex constants. Then (4) is a WGBF for $L^{2}(S, \mathbb{C}^{2})$ related to $L_{c}^{\infty }(S, \mathbb{C}^{2})$, but it is not a GBF for $L^{2}(S, \mathbb{C}^{2})$.

### Proof

By a simple argument, **g**, **h** satisfy the assumptions of Example 4.1. So (4) is a WGBF for $L^{2}(S, \mathbb{C}^{2})$ related to $L_{c}^{\infty }(S, \mathbb{C}^{2})$. Observe that at least one of $\sum_{n\in \mathbb{Z}}\vert g_{1}(\cdot -na) \vert ^{2}$, $\sum_{n\in \mathbb{Z}}\vert {g_{2}(\cdot -na)} \vert ^{2}$, $\sum_{n\in \mathbb{Z}}\vert {h_{1}(\cdot -na)} \vert ^{2}$ and $\sum_{n\in \mathbb{Z}}\vert {h_{2}(\cdot -na)} \vert ^{2}\notin L^{\infty }( \mathbb{R})$. Then at least one of $\mathcal{G}({g_{1}}, a, b)$, $\mathcal{G}({g_{2}}, a, b)$, $\mathcal{G}({h_{1}}, a, b)$ and $\mathcal{G}({h_{2}}, a, b)$ is not a Bessel sequence in $L^{2}( \mathbb{R})$ by [35], Proposition 11.3.4. It follows that at least one of $\mathcal{G}(\mathbf{g}, a, b)$ and $\mathcal{G}( \mathbf{h}, a, b)$ is not a Bessel sequence in $L^{2}(S, \mathbb{C}^{2})$. This shows that (4) is not a GBF for $L^{2}(S, \mathbb{C}^{2})$. □

## Conclusions

The construction of bi-frames is a fundamental problem in frame theory. In recent years, the study of vector-valued frames and subspace frames has interested many mathematicians due to their wide applications. The concept of weak bi-frame generalizes that of bi-frame, and it has potentials in broadening applications of the frame theory in computation. We in this paper introduce the WGBF under the setting of vector-valued subspaces, characterize WGBFs on the time domain, and present some examples. Our result generalizes that of scalar-valued functions in the literature. Due to the more complicated geometry of inner products in vector-valued function spaces than in scalar-valued function spaces, our result is not a trivial generalization.
